# Onychophoran Hox genes and the evolution of arthropod Hox gene expression

**DOI:** 10.1186/1742-9994-11-22

**Published:** 2014-03-05

**Authors:** Ralf Janssen, Bo Joakim Eriksson, Noel N Tait, Graham E Budd

**Affiliations:** 1 Department of Earth Sciences, Palaeobiology, Uppsala University, Villavägen 16, 75236 Uppsala, Sweden; 2 Department of Neurobiology, University of Vienna, Althanstrasse 14, 1090 Vienna, Austria; 3 Department of Biological Sciences, Macquarie University, Sydney NSW, 2109, Australia

**Keywords:** Development, Segmentation, Body patterning, Phylogeny, Tagmosis

## Abstract

**Introduction:**

Onychophora is a relatively small phylum within Ecdysozoa, and is considered to be the sister group to Arthropoda. Compared to the arthropods, that have radiated into countless divergent forms, the onychophoran body plan is overall comparably simple and does not display much in-phylum variation. An important component of arthropod morphological diversity consists of variation of tagmosis, i.e. the grouping of segments into functional units (tagmata), and this in turn is correlated with differences in expression patterns of the Hox genes. How these genes are expressed in the simpler onychophorans, the subject of this paper, would therefore be of interest in understanding their subsequent evolution in the arthropods, especially if an argument can be made for the onychophoran system broadly reflecting the ancestral state in the arthropods.

**Results:**

The sequences and embryonic expression patterns of the complete set of ten Hox genes of an onychophoran (*Euperipatoides kanangrensis*) are described for the first time. We find that they are all expressed in characteristic patterns that suggest a function as classical Hox genes. The onychophoran Hox genes obey spatial colinearity, and with the exception of *Ultrabithorax* (*Ubx*), they all have different and distinct anterior expression borders. Notably, *Ubx* transcripts form a posterior to anterior gradient in the onychophoran trunk. Expression of all onychophoran Hox genes extends continuously from their anterior border to the rear end of the embryo.

**Conclusions:**

The spatial expression pattern of the onychophoran Hox genes may contribute to a combinatorial Hox code that is involved in giving each segment its identity. This patterning of segments in the uniform trunk, however, apparently predates the evolution of distinct segmental differences in external morphology seen in arthropods. The gradient-like expression of *Ubx* may give posterior segments their specific identity, even though they otherwise express the same set of Hox genes. We suggest that the confined domains of Hox gene expression seen in arthropods evolved from an ancestral onychophoran-like Hox gene pattern. Reconstruction of the ancestral arthropod Hox pattern and comparison with the patterns in the different arthropod classes reveals phylogenetic support for Mandibulata and Tetraconata, but not Myriochelata and Atelocerata.

## Introduction

Arthropod segmentation – its origin and maintenance - is among the key topics of evolutionary developmental (Evo-Devo) research. Arthropods are of particular interest as they comprise the most speciose and disparate animal phylum. They form a clade together with the onychophorans and tardigrades, referred to as Panarthropoda [1]. The arthropods themselves include four or five (depending on the status of pygnogonids) classes [2,3]: the insects, the crustaceans, the myriapods and the chelicerates (with or without the pycnogonids), although there is significant molecular support for insects being an ingroup to a paraphyletic “Crustacea”, the group in total being referred to as the Pancrustacea or Tetraconata [3,4]. While the body plans of many arthropod groups have diversified significantly since the Cambrian Period, the lobopodian body plan, represented today by the onychophorans, appears to have changed relatively little [5-8]. Many of the key characteristics of the arthropods - such as jointed limbs and full adult body segmentation with pronounced segmental indentations - are not present in onychophorans, and, based on reconstructions of the arthropod stem-group, these absences are best interpreted as being primitive [9]. However, traces of such features, such as the genetic toolkit required for podomere patterning are present in the limbs of onychophorans [10], and arthropod-like appendages are present in stem-group arthropods such as the anomalocaridids [11,12].

In *Drosophila* and to some extent in other arthropods, segmentation is under control of a hierarchic segmentation gene cascade reviewed in [13-15]. This cascade controls the expression of the Hox genes, which in turn specify segmental identity. It is believed that the Hox genes are involved in providing positional information in a combinatorial mode to give each segment its identity along the anterior-posterior body axis [16-19]. Thus, disturbance of the Hox patterning, such as loss-of-function or ectopic expression of Hox genes often results in homeotic transformations, the change of one segment’s identity into that of another [20-23]. Beyond that, Hox genes are believed to be involved in tagmosis, i.e. the grouping of segments into functional units (tagmata) e.g. [24,25]. The *Drosophila* Hox clusters contain eight Hox genes, but the ancestral arthropod Hox cluster most likely contained ten Hox genes [26,27]. Two of the *Drosophila* genes, however, have changed their function. These are *fushi-tarazu* (*ftz*) and *Hox3.* The latter gene evolved into *bicoid* (*bcd*), *zerknüllt* (*zen*), (also referred to as *z1*) and *z2*[28,29]. These genes have lost their homeotic function and now have new expression patterns. In *Drosophila, ftz* acts as a pair rule gene [30], and *bcd*/*zen*-genes are involved in axis determination and formation of extraembryonic membranes [31]. However, in more basally branching arthropods, the expression patterns of *Hox3* and *ftz* are consistent with canonical Hox-like domains [32-36].

As Hox gene expression in arthropods is quite diverse (reviewed in e.g. [27]), it is difficult to reconstruct the ancestral pattern of expression within the clade without reference to an outgroup, such as the onychophorans [37-39].

Here we report on the sequences and embryonic gene expression profiles of the complete set of the ten Hox genes in the onychophoran *Euperipatoides kanangrensis*. The new data contribute to our understanding of how the highly derived arthropod body plans have evolved from a rather uniform onychophoran-like ancestor. Based on the onychophoran data we reconstruct the ancestral arthropod Hox gene profile as far as possible and use this in a comparative approach to detect phylogenetic signal. We find support for Mandibulata and Tetraconata, but not Myriochelata and Atelocerata.

## Material and methods

### Species husbandry, embryo treatment, in situ hybridization, nuclei staining and data documentation

The collection, laboratory maintenance and dissection of embryos of *Euperipatoides kanangrensis* specimens are described in [40]. Whole mount *in situ* hybridization was performed as described in [10]. The developmental stage of all embryos was determined by analyzing embryos stained with the nuclear dye DAPI (4-6-Diamidino-2-phenylindole) and comparing to the stage table published by [41]. Embryos were analyzed under a Leica dissection microscope equipped with either an Axiocam (Zeiss) or a Leica DC100 digital camera. Brightness, contrast, and color values were adjusted in all images using the image processing software Adobe Photoshop CS2 (Version 9.0.1 for Apple Macintosh).

### RT-PCR and gene cloning

Total RNA was isolated from freshly-dissected embryos of *E. kanangrensis* via TRIZOL (Invitrogen). Poly-A RNA was isolated with the PolyATtract mRNA isolation system III (Promega) and used to produce cDNA using the Superscript first strand synthesis system for RT-PCR (Invitrogen). Short fragments of the Hox gene orthologs of *E. kanangrensis Sex combs reduced* (*Ek-Scr*), *fushi-tarazu* (*Ek-ftz*) and *Abdominal-B* (*Ek-Abd-B*) were isolated via RT-PCR with degenerate primers. For that purpose mRNA was isolated and cDNA was synthesized from complete embryos representing all stages from the 1-cell stage up to stage 21 [41]. A list of the primers used is available from the authors upon request. Initial PCR fragments were elongated via 3′-RACE (for *Ek-Scr*, *Ek-ftz* and *Ek-Abd-B*) or 5′-RACE (for *Ek-Antp*) with the GENE RACER KIT (Invitrogen) to obtain sufficiently long fragments for subsequent *in situ* hybridization experiments. These sequences are available in GenBank under the accession numbers FR865437 (*Ek-Scr*), FR865438 (*Ek-ftz*), FR865439 (*Ek-Antp*), and FR865440 (*Ek-Abd-B*). We also screened two independently prepared embryonic transcriptomes of *E. kanangrensis* for the presence of Hox genes and found a single copy of each of the expected Hox gene orthologs. The embryonic transcriptomes were made from comparable stages as used for the RT-PCR screen (1-cell stage to stage 21) [41]. We discovered the complete open reading frames of all onychophoran Hox genes. These sequences are available under accession numbers HE979835 (*Ek-lab*), HE979836 (*Ek-pb*), HE979837 (*Ek-Hox3*), HE979838 (*Ek-Dfd*), HE979839 (*Ek-Scr*), HE979840 (*Ek-ftz*), HE979841 (*Ek-Antp*), HE979842 (*Ek-Ubx*), HE979843 (*Ek-abd-A_splice variant I*), HE979844 (*Ek-abd-A_splice variant II*), and HE979845 (*Abd-B*).

All fragments were cloned into the PCRII vector (Invitrogen). Sequences of all isolated fragments were determined from both strands by means of Big Dye chemistry on an ABI3730XL analyser by a commercial sequencing service (Macrogen, Korea).

### Phylogenetic analysis

The complete homeodomains were aligned in Clustal_X [42,43] and accuracy of the resulting alignment was checked by hand. Maximum likelihood analysis was performed using the LG substitution model [44] as implemented in PhyLM [45].

### Segmental nomenclature

In order to facilitate description and comparison of the data, we label segments that express Hox genes in arthropods with the prefix “H”; H1 is thus the most anterior segment (corresponding variously to the onychophoran slime papilla segment, chelicerate pedipalps, insect and myriapod “intercalary” and crustacean second antenna segments) that expresses Hox genes (i.e. *lab*, *pb* and *Hox3*), thus avoiding the potentially thorny problem of how many segments lie in front of this. Onychophoran segments in general are labelled fap (frontal appendage), j (jaw), sp (slime papilla) and then L1-15 for the walking limb-bearing segments. L1 is thus H2.

## Results

### Sequence analysis of the ten onychophoran Hox genes

Partial sequences of the *E. kanangrensis* Hox gene orthologs *Ek-lab*, *Ek-pb*, *Ek-Hox3*, *Ek-Dfd*, and *Ek-Ubx* have been described in [40,46]. We have now recovered the complete protein coding sequences of the ten onychophoran Hox genes. Orthology of the complete protein sequences of the *E. kanangrensis* Hox genes was determined by comparison with published orthologs of the beetle *Tribolium castaneum*. The published sequence of *ftz* in the sea spider *Endeis spinosa* was added to the analysis to reveal orthology of *ftz* genes (Additional file 1: Figure S1). We provide the alignment of conserved regions of the Hox genes from various arthropod and onychophoran species (see supplementary data for further information). Within the highly conserved regions lie diagnostic amino acids that are characteristic for each Hox gene (Additional file 2: Figure S2). We did not detect any duplicated Hox genes, neither in our PCR screens nor in the sequenced transcriptomes although two *Abd-B* paralogs have been reported for another onychophoran species, *Akanthokara kaputensis* (accession numbers AF011273 (*Abd-B-1*) and AF011274 (*Abd-B-2*)). Interestingly, we found one splice variant of *Ek-abd-A* with a hexapeptide (HX) sequence (*Ek-abd-A*-variant I), and one without this highly-conserved motif (*Ek-abd-A*-variant II). For all other onychophoran Hox genes we only identified transcripts with the HX motif (except for *Ek-Abd-B*, which generally lacks a HX motif) (Additional file 2: Figure S2). This result differs from the earlier published fragment of *Ek-Dfd*[40], which lacks a HX motif and also differs considerably in its complete N-terminal region from the newly recovered fragment of *Ek-Dfd*. The newly recovered fragment has significant sequence similarity with *Dfd* genes of other arthropods (Additional file 2: Figure S2). The previously published short fragment of *Akanthokara Dfd*[47] most probably represents an insect sequence since it contains a number of insect-specific amino acids (Additional file 2: Figure S2). The earlier published sequence of *Ek-Hox3*[40] also differs from the newly-recovered fragment. The former sequence contains a short string of additional amino acids between the HX motif and the homeodomain (HD). This sequence was found neither in the sequenced transcriptomes nor in newly-cloned fragments recovered by means of RT-PCR with gene specific primers that flanked the sequence in question (not shown). It remains unclear, however, whether the former sequence represents an artefact or a rare splice variant.

### Expression of the ten onychophoran Hox genes

**Figure 1:**
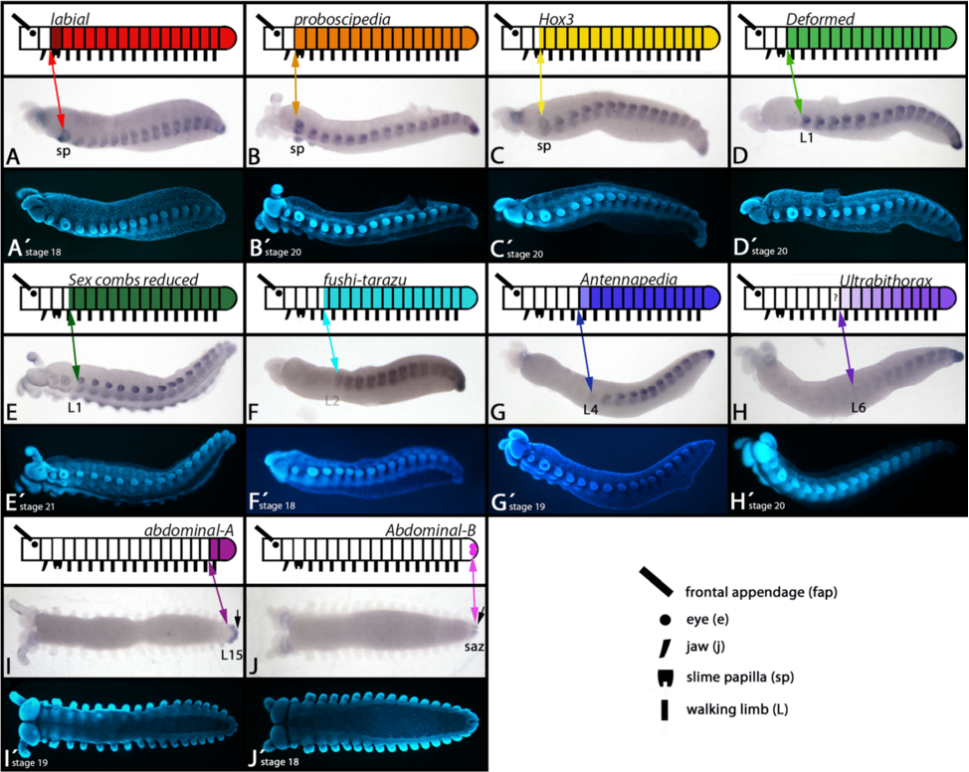
**Expression of onychophoran Hox genes (lateral/ventral views). (A-H)** Lateral views. **(I-J)** Ventral views. In all cases anterior is to the left. Upper row: schematic drawing of Hox gene expression. Middle row: bright-field photography **(A-J)**. Lower row: DAPI staining **(A’-J’)** of the same embryos as shown in exactly the same position as in the middle row **(A-J)**. Darker shading in schematic drawings indicates stronger expression. Lighter shading indicates weaker expression. Note that the expression of *Ek-Ubx* mRNA forms a posterior to anterior gradient with unclear anterior expansion (marked with a question mark (?)). Arrows in **I-J** point to expression in the anal valves. Abbreviations: e, eye; j, jaw; L1-L15, first to fifteenth walking limb; saz, segment addition zone; sp, slime papilla.

**Figure 2:**
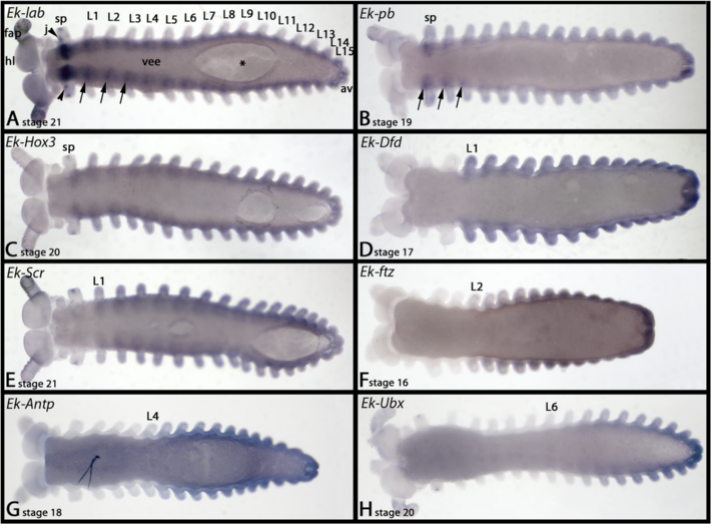
**Expression of onychophoran Hox genes (ventral views).** In all cases anterior is to the left. Arrows in **A** and **B** point to up-regulated expression of *lab* and *pb* in the ventral nervous system. The asterisk (*) in A indicates damage of ventral tissue as a result of the hybridization procedure. See text for further information. Abbreviations; av, anal valves; fap, frontal appendage; hl, head lobe; j, jaw, L1-L15, first to fifteenth walking limb; sp, lime papilla, vee, ventral extraembryonic ectoderm.

The expression profiles of *Ek-lab*, *Ek-pb*, *Ek-Hox3* and *Ek-Dfd* have been described in [40] (Figures 1A-D and 2A-D). The expressions of all ten Hox genes are continuous from their respective anterior borders of expression back to the rear of the developing embryos.

#### labial

In addition to the previously-described expression we find that *Ek-lab* is expressed considerably more strongly in the slime papillae-bearing (sp) segment (Figures 1A and 2A) as compared to other segments, and expression is up-regulated in certain regions of the developing neuroectoderm of the trunk (Figure 2A). A spot of *Ek-lab* expression is located anteriorly and proximally in the slime papillae (Figure 2A).

#### proboscipedia

The only difference between the published expression pattern and our new data is that *Ek-pb* is up-regulated in some regions of the neuroectoderm (Figure 2B).

#### Hox3

*Ek-Hox3* remains expressed in the posterior half of the slime papillae, and does not later disappear from this tissue (Figure 2C) cf. [40].

#### Deformed

The anterior border of *Ek-Dfd* is at the border between the jaw-bearing segment and the first trunk segment (L1) (Figures 1D and 2D). In embryos younger than stage 11, expression only extends into L2 (Additional file 3: Figure S3).

#### Sex combs reduced

**Figure 3:**
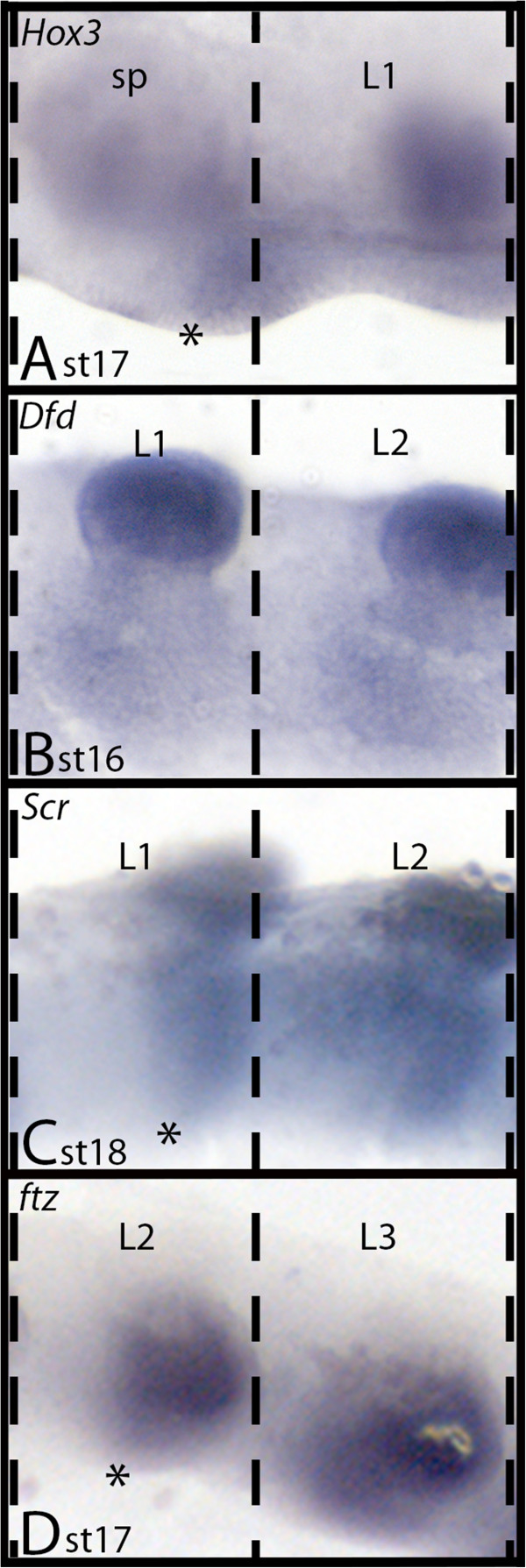
**Anterior borders of *Ek-Hox3*, *Ek-Scr, Ek-Dfd *and *Ek-ftz *expression. A)***Ek-Hox3* expression in sp and L1. Asterisk marks anterior border of expression in the middle of the slime papilla (sp) segment (cf. Eriksson et al. 2010). **B)** Expression of *Ek-Dfd* extends to the anterior of the L1 segment (cf. Eriksson et al. 2010). **C)***Ek-Scr*. Asterisk marks anterior border of expression in the middle of the L1 segment. **D)***Ek-ftz*. Asterisk marks anterior border of expression in the middle of the L2 segment. Abbreviations as in Figure 1. Dashed lines indicate segmental borders.

The anterior border of *Ek-Scr* expression lies approximately in the middle of the first walking limb-bearing segment (L1) (Figures 1E, 2E and 3). From there the expression extends throughout the complete posterior body including the anal valves.

#### fushi-tarazu

The anterior border of *Ek-ftz* lies in the middle of the second trunk segment (L2) (Figures 1F, 2F and 3). In embryos younger than stage 12, expression only extends anteriorly into L3 (Additional file 3: Figure S3).

#### Antennapedia

*Ek-Antp* is expressed in all posterior segments including all of L4. Expression in L4, however, is considerably weaker than in more posterior segments. In segments L5-L7, *Ek-Antp* is expressed stronger than in L4, but still slightly weaker than in L8 to L15, which is the last segment (Figures 1G and 2G). Whether expression in L5 to L7 forms a gradient, or if it is at the same level in these segments, is not revealed by our *in situ* hybridization technique. As described for *Scr* and *ftz*, expression of *Antp* also shifts towards the anterior at around stage 13 (Additional file 3: Figure S3).

#### Ultrabithorax

The *Ek-Ubx* gene is clearly expressed in an anterior to posterior gradient. The most anterior segment expressing *Ek-Ubx* at a detectable level is L6 (Figures 1H and 2H). The gradient makes it difficult to determine unambiguously the anterior-most extent of *Ek-Ubx* expression. Low-level transcription (below detectable range) may be present in L5.

#### abdominal-A

*Ek-abd-A* is exclusively expressed in the last trunk segment (L15) and the anal valves (Figure 1I). Notably, expression in the mesoderm is stronger than in the ectoderm and at late developmental stages *Ek-abd-A* is strongly expressed in the interface between L15 and the anal valves (Additional file 4: Figure S4). Note the difference between this specific staining and frequently occurring unspecific signal in L15 (Additional file 5: Figure S5).

#### Abdominal-B

*Ek-Abd-B* is expressed in the mesoderm of the entire anal valves, but ectodermal expression is restricted to the very posterior of the anal valves (Figure 1J and Additional file 4: Figure S4).

Generally, none of the ten onychophoran Hox genes are expressed in tissue dorsal to the base of the appendages (Figure 1). This is unlike the situation in arthropods where expression of the Hox genes is generally not restricted to ventral tissue e.g. [27,34,48].

## Discussion

### Comparison of arthropod and onychophoran Hox gene expression data

**Figure 4:**
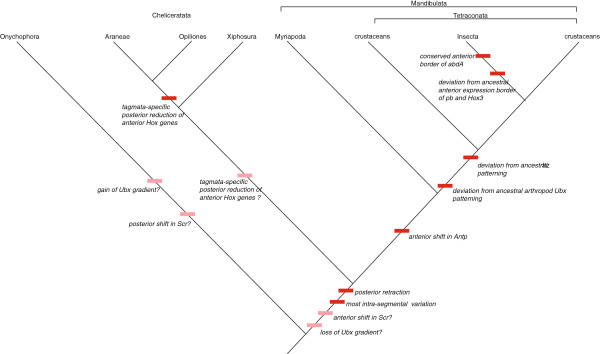
**Summary of phylogenetic signals based on onychophoran and arthropod Hox gene expression.** Conserved and diverged characters are plotted on the nowadays best-supported arthropod phylogeny. Onychophora, and onychophoran data, serve as outgroup. Dark red boxes denote events during arthropod Hox gene evolution. Light red boxes denote unresolved character-state; in these cases the character-state cannot be determined ancestral or derived because outgroup data for a clade Onychophora + Arthropoda are not available.

Although the expression patterns of the Hox genes are relatively conserved across the arthropods, some clade-specific differences exist [27]. Comparison between arthropod and onychophoran Hox gene expression patterns may aid in reconstructing ancestral and derived arthropod expression: firstly, shared features of onychophorans and arthropods are likely to have been present in the last common ancestor of both; and secondly, the onychophoran data can in some instances be used to polarise the order of Hox gene change within the arthropod tree itself. We have therefore mapped Hox gene expression patterns onto an arthropod plus onychophoran tree (Figure 4). The Mandibulata grouping (myriapods plus insects plus crustaceans) seems favoured over Paradoxopoda (myriapods and chelicerates) in recent phylogenies [49-53] and thus we have chosen this topology to map our data onto, although as noted below, some aspects of the onychophoran data do support Mandibulata and none support Paradoxopoda.

Onychophorans and arthropods share several important aspects of their Hox gene expression patterns. Firstly, in both onychophorans and arthropods, Hox gene expression is absent from tissue anterior to the slime papillae-bearing (intercalary/premandibulary/pedipalpal) segment [25,34,40,54]: i.e., Hox gene expression never extends to the anterior-most segments in either clade. Secondly, all Hox genes, except onychophoran *Ubx*, have a distinct anterior expression border, although at early developmental stages in members of both phyla this border may be located one segment more posteriorly.

**Figure 5:**
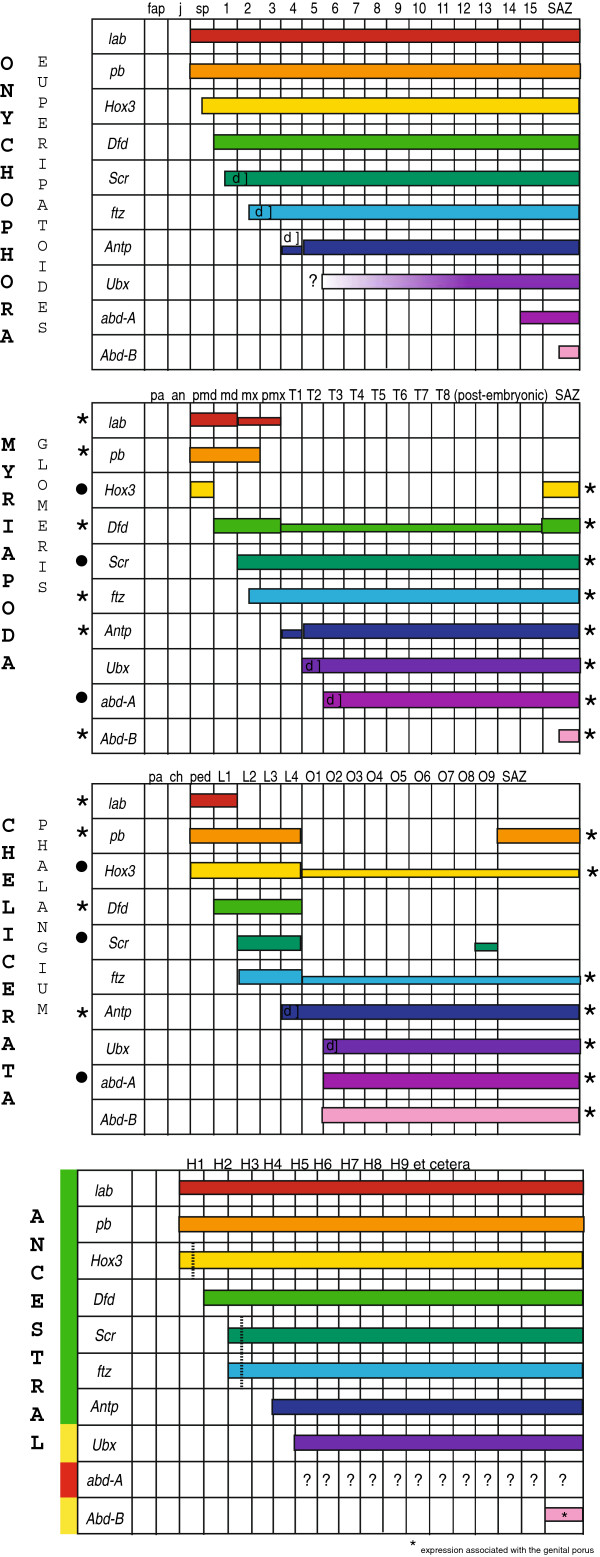
**Comparison of Hox gene expression profiles in the onychophoran *E. kanangrensis*, the myriapod *Glomeris marginata *and the harvestman *Phalangium opilio, and *reconstruction of the putative Hox gene pattern in the common ancestor.** Orthologous genes are with identical colours. Conserved anterior and posterior borders of expression (compared to *E. kanangrensis*) are marked with asterisks (*). Borders of expression that are conserved in the myriapod and the harvestman (but not the onychophoran) are marked with filled circles (•). Smaller bars indicate weaker expression. ‘Delayed’ expression is marked with ‘d]’. Green bar to the left indicates good support for the predicted corresponding expression patterns in the ancestor; yellow bars indicate ambiguous support for the reconstructed expression patterns; red bar indicates that the ancestral expression pattern of *abd-A* is not predictable. This is also indicated by question marks (?). Dashed vertical lines indicate an either segmental or parasegmental (dashed line) anterior expression border. Hox gene-expressing segments are labelled with the prefix ‘H’. Abbreviations are as in Figure 1, and an, antennal segment; ch, cheliceral segment; L1-L4 (in chelicerates), walking limb-bearing segments one to four; md, mandibular segment; mx, maxillary segment; O1-O9, first to ninth opisthosomal segment; pa, pre-antennal region; ped, pedipalpal segment; pmd, premandibular segment; pmx, postmaxillary segment; SAZ, segment addition zone; T1-T8, first to eighth walking limb-bearing segment.

A difference between onychophorans and arthropods is that in arthropods *Scr* is expressed in H3, but in onychophorans it is in H2. It would appear that either the onychophoran pattern is derived, or that a shift in the anterior extension of *Scr* evolved in the lineage leading to the arthropods (Figures 4 and 5 and Additional file 6: Table S1). Another apparent difference concerns the posterior extent of gene expression. In *E. kanangrensis* the expression of all Hox genes extends back to the rear of the embryo [40], this study; but in arthropods, the “anterior” Hox genes are typically not expressed in the posterior part of the body. However, data on Hox gene expression in species of the Chelicerata and Myriapoda provide evidence that the onychophoran Hox gene pattern may in this respect reflect the ancestral condition for Onychophora + Arthropoda. In the harvestman *Phalangium opilio*[36] (Figure 5A), the spider *Achaearanea tepidariorum*[55], the mite *Archegozetes longisetosus*[56], the centipede *Lithobius atkinsoni*[54] and the millipede *Glomeris marginata*[34] (Figure 5A), the expression of at least some anterior Hox genes extends from their anterior border of expression to the rear end of the embryo. In other cases anterior Hox genes are also expressed in the most posterior segment(s) and/or the segment addition zone (SAZ) (Figure 5A). We believe that weak (but clearly detectable) expression of some Hox genes in the posterior segments and expression in the last segment (or the SAZ) may represent evolutionary remnants of an ancestral state more clearly retained in onychophorans. Taken together, these data indicate a tendency in arthropod evolution to restrict anterior Hox gene expression to distinct anterior regions.

### Clade-specific comparisons between onychophorans and arthropods

As well as the general similarities (and differences) highlighted above, onychophorans share several similarities at the arthropod clade-specific level, in particular in conservation of anterior Hox gene expression borders (summarised in Figure 4). Although the anterior Hox gene pattern of *pb and Hox3* is highly conserved, it deviates in some clades at least in insects, and these latter seem therefore to be derived. Secondly, deviation from the pattern of *ftz* expression found in onychophorans is only found in crustaceans and insects, supporting the Tetraconata (Pancrustacea) hypothesis. Thirdly, the anterior border of *Antp* seen in onychophorans has switched from H5 to H4 in myriapods, crustaceans and insects, in agreement with the Mandibulata concept. Fourthly, the posterior reduction of expression of the anterior Hox genes discussed above progresses from onychophorans, where expression of all the Hox genes extend to the rear end, to crustaceans and insects, where expression is more confined to distinct anterior regions (Figure 5A and Figure twelve in [27]). This again is in agreement with the Tetraconata concept. Conversely, the polarisation possible with the onychophoran data does not support either Myriochelata or Atelocerata.

Taken together, the differences and similarities in the expression data between onychophorans and arthropods allow a reasonable degree of reconstruction of the ancestral Hox expression for the entire clade (Figure 5B). We find good support for the reconstruction of the pattern of anterior Hox genes (*lab*, *pb*, *Hox3*, *Dfd*, *Scr*, *Antp*), but also that the reconstruction of posterior Hox genes is difficult (*Ubx*, *abd-A*, *Abd-B*) (Figure 5B). The varying anterior border of *abd-A* in the onychophoran and various arthropods makes it impossible to reconstruct its ancestral pattern. The lability of *abd-A* is highlighted by the loss of this gene from e.g. the mite *Tetranychus urticae*[57]. Among the reconstructable patterns some uncertainties remain, such as whether the *Ubx* gradient in onychophorans is ancestral or derived, and the anterior expression boundary of *Scr*. These uncertainties might in principle be resolved by reference to a suitable outgroup such as a tardigrade or a cycloneuralian worm. Unfortunately, Hox gene expression and action in the nematode *Caenorhabditis elegans* is highly derived [58], and no other cycloneuralian expression patterns are known. Development of a priapulid *in situ* hybridization protocol [59], however, opens the possibility of such data being obtained in the future.

### Hox gene expression and tagmosis in arthropods

Hox gene expression has long been associated with the evolution and maintenance of tagmosis, i.e. the characteristic grouping or fusion of functionally similar segments e.g. [24,25,36,60]. Ever since the earliest days of Hox gene research, the dominant hypothesis has been that the Hox genes primarily act to specify regions of the body, including above all the tagmata [16].

Although onychophorans have a relatively unspecialised body plan, with only the head and most posterior segment being differentiated, their Hox gene expression patterns show a surprising degree of sophistication, for example in the gradient of expression of *Ubx*. This pattern mirrors a similar one documented in limb specification, which in onychophorans is also surprisingly sophisticated [10].

In the onychophoran studied, the anterior segments (sp-L5) and the last segment (L15) express a unique set of Hox genes, which allows for the characterization of each of these segments *sensu*[17] (Figure 5A). The posterior segments (L6-L14), however, all express the same eight Hox genes. The same is true for many arthropods where the posterior segments are usually not characterised by a unique set of Hox genes [27]. It is possible that posterior segments in onychophorans do not require a specific set of Hox gene input as they appear to be morphologically identical, but that would then raise the question why the anterior (morphologically identical) trunk segments in onychophorans do express distinct sets of Hox genes (Figure 5A). It is of course possible that there are some cryptic morphological differences in these segments that are being regulated by differential Hox gene expression. However, if this is the case, then it is possible that such cryptic morphological differences are present in the posterior segments too, and this would raise the question of how these segments are specified within identical Hox gene expression domains. Generally, the mRNA expression pattern can differ from the protein pattern as a result of translational repression, and this could lead to different Hox-protein and Hox-mRNA landscapes. Further differences in segmental Hox gene patterning might also involve Hox cofactors [61-63], the presence of alternative splice variants e.g. [64] (possibly as represented by the different splice variants of *Ek-abd-A*), and temporal differences in Hox gene expression [65,66]. Such temporal differences include the shifting anterior expression borders of *Ek-Scr*, *Ek-ftz*, and *Ek-Antp* (Additional file 3: Figure S3).

*Ek-Ubx* mRNA is expressed in an anterior to posterior gradient with detectable transcripts from the very posterior of the embryo to at least L6. It is thus possible that the different levels of *Ek-Ubx* mRNA (and resulting *Ek*-Ubx protein) are sufficient to give these segments a unique Hox signature. Studies in at least *Drosophila* do indeed show that *Ubx* function in segmentation and limb development is dependent on different levels of expression [67,68]. Similarly, in the crustacean *Parhyale*, different levels of *Ubx* expression may be responsible for the development of different types of appendages, and knock down of *Ubx* results in homeotic transformation of these segments [69]. The *Ek-Ubx* gradient thus offers one possibility of how each posterior segment is uniquely patterned, even though they express the same set of Hox genes.

## Competing interests

The authors declare that they have no competing interests.

## Authors’ contributions

RJ designed the project, carried out the experiments and wrote the first draft of the manuscript. BJE isolated fragments of some Hox genes prior to the availability of sequenced transcriptomes, and provided additional transcriptome data. NNT provided interpretation and discussion and was involved in writing of the final version of the manuscript. He also identified the species. GB provided interpretation and discussion and was involved in writing of the final version of the manuscript. All authors read and accepted the final manuscript.

## Supplementary Material

Additional file 1: Figure S1 Phylogenetic analysis of *E. kanangrensis* Hox genes. The homeodomain sequences have been used for the analyses. The phylogram represents the unrooted majority rule consensus computed from 100 replicas. Numbers indicate the reliability values of the branches. Click here for file

Additional file 2: Figure S2 Conserved protein motifs in arthropod and onychophoran Hox genes. Onychophoran Hox sequences (*E. kanangrensis* and *Akanthokara kaputensis*) are aligned with orthologous genes of at least one species representing the four main arthropod classes (i.e. Insecta, Crustacea, Myriapoda and Chelicerata). Identical amino acids (compared to *E. kanangrensis*) are shaded in grey; amino acids that are conserved in arthropods are shaded in yellow; diagnostic amino acids are shaded in red. Amino acids in red font indicate insect-specific positions (see text for further information). Question marks (?) stand for unknown sequence. Dashes (-) indicate the absence of any amino acid in this position of the alignment. Sequences of *E. kanangrensis* are underlined. Asterisks (*) mark *G. marginata* sequences that are partially recovered from an embryonic transcriptome. Abbreviations: *Af*, *Artemia franciscana* (Crustacea); *Ak*, *Akanthokara kaputensis* (Onychophora); *At*, *Achaearanea tepidariorum* (Chelicerata); *Cs*, *Cupiennius salei* (Chelicerata); *Dm*, *Daphnia magna* (Crustacea) *Dp*, *Daphnia pulex* (Crustacea); *Ek*, *Euperipatoides kanangrensis* (Onychophora); *Glomeris marginata* (Myriapoda); *Mb*, *Mamestra brassicae* (Insecta); *Sm*, *Strigamia maritima* (Myriapoda); *Tc*, *Tribolium castaneum* (Insecta). Click here for file

Additional file 3: Figure S3 Shifted anterior borders of Hox gene expression. All embryos are oriented with anterior to the left. A/B: Ventral view. C-H: lateral view. **A-H)** bright field photographs; **A’-H’)** DAPI staining of the same embryos shown in A-H. Double-headed arrows point to anterior border of expression. Faint arrows indicate weak expression. **A)** Expression of *Ek-Dfd* is anteriorly restricted to L2 (stage 9/10). **B)** At stage 11 *Ek-Dfd* expression extends to the anterior border of L1. **C)** Expression of *Ek-Scr* extends into the middle of L2 at stage 12. **D)** At stage 13 *Ek-Scr* shifts towards anterior into the L1-segment. **E)** Anterior border of expression of *Ek-ftz* is in the middle of L3 at stage 11. **F)** At the subsequent stage 12, *Ek-ftz* is weakly present also in L2. **G)** Expression of *Ek-Antp* is restricted to L5 (and more posterior segments) at stage 12. **H)** At stage 13, *Ek-Antp* reaches anteriorly into the L4 segment where it remains expressed weakly during further development. Shifting of the anterior border of expression was never observed for any of the other onychophoran Hox genes. Abbreviations: L1-L5, first to fifth walking limb-bearing segment. Click here for file

Additional file 4: Figure S4 Additional aspects of *Ek-abd-A* and *Ek-Abd-B* expression. In all panels anterior is to the left. **A)** Expression of *Ek-abd-A*. Ventral view. Expression in the mesoderm (mes) is stronger than in the ectoderm (ec). **B)** Expression of *Ek-abd-A*; dorsal view. **C)** Expression of *Ek-Abd-B.* Ventral view. The arrow points to expression in the ectoderm of the anal valves. Abbreviations: a, anus; av, anal valve; ec, ectoderm; mes, mesoderm; L14 and L15, fourteenth and fifteenth walking-limb bearing segment. Click here for file

Additional file 5: Figure S5 Frequently occurring staining artefacts. In all panels anterior is to the left. **A)** Stage 19 embryo. Ventral view. Typical staining artefacts are often observed in older stage embryos where the surface of the frontal appendages attracts unspecific signal. A similar false positive signal appears regularly in the opening of the slime papillae. The coelomic cavity of the L15-segment often stains unspecifically. Long staining time frequently results in unspecific staining of the yolk. **B/C)** A typical artefact: staining of one of the two coelomic cavities (arrowheads) of the head in a stage 13 and a stage 20 embryo. Abbreviations: fap, frontal appendage; L15, fifteenth walking limb; sp, lime papilla. Click here for file

Additional file 6: Table S1 Summary of available literature on Hox gene expression in arthropods and onychophorans. Compared is the anterior-most extension of Hox gene mRNA (in some cases protein) expression. Click here for file

## References

[B1] Campbell LI Rota-Stabelli O Edgecombe GD Marchioro T Longhorn SJ Telford MJ Philippe H Rebecchi L Peterson KJ Pisani D MicroRNAs and phylogenomics resolve the relationships of Tardigrada and suggest that velvet worms are the sister group of Arthropoda Proc Natl Acad Sci USA 2011 108 15920 15924 10.1073/pnas.1105499108 21896763 PMC3179045

[B2] Dunlop JA Arango CP Pycnogonid affinities: a review J Zool Syst Evol Res 2005 43 8 21 10.1111/j.1439-0469.2004.00284.x

[B3] Giribet G Edgecombe GD Reevaluating the arthropod tree of life Annu Rev Entomol 2012 57 167 186 10.1146/annurev-ento-120710-100659 21910637

[B4] Dohle W Are the insects terrestrial crustaceans? A discussion of some new facts and arguments and the proposal of the proper name “Tetraconata” for the monophyletic unit Crustacea + Hexapoda Ann Soc Entomol Fr 2001 37 85 103

[B5] Whittington HB The lobopod animal *Aysheaia pedunculata* Walcott, Middle Cambrian, Burgess Shale, British Columbia Philos Trans R Soc Lond B Biol Sci 1978 284 165 197 10.1098/rstb.1978.0061

[B6] Thompson I Jones DS A possible onychophoran from the Middle Pennsylvanian Mazon Creek Beds of northern Illinois J Paleontol 1980 54 588 596

[B7] Dzik J Krumbiegel G The oldest ‘onychophoran’ Xenusion: a link connecting phyla? Lethaia 1989 22 169 181 10.1111/j.1502-3931.1989.tb01679.x

[B8] Ou Q Liu J Shu D Han J Zhang Z Wan X Lei Q A rare onychophoran-like lobopodian from the Lower Cambrian Chengjiang Lagerstätte, Southwest China, and its phylogenetic implications J Paleontol 2011 85 587 594 10.1666/09-147R2.1

[B9] Budd GE Tardigrades as ‘stem-group arthropods’: the evidence from the Cambrian fauna Zool Anz 2001 240 265 279 10.1078/0044-5231-00034

[B10] Janssen R Eriksson BJ Budd GE Akam M Prpic NM Gene expression patterns in an onychophoran reveal that regionalization predates limb segmentation in pan-arthropods Evol Dev 2010 12 363 372 10.1111/j.1525-142X.2010.00423.x 20618432

[B11] Daley AC Budd GE Caron JB Edgecombe GD Collins D The Burgess Shale anomalocaridid *Hurdia* and its significance for early euarthropod evolution Science 2009 323 1597 1600 10.1126/science.1169514 19299617

[B12] Daley AC Budd GE New anomalocaridid appendages from the Burgess Shale, Canada Palaeontology 2010 53 721 738 10.1111/j.1475-4983.2010.00955.x

[B13] Klingler M Tautz D Russo VEA, Cove DJ, Edgar LG, Jaenisch R, Salamini F Formation of embryonic axes and blastoderm pattern in *Drosophila* Development, Genetics, Epigenetics and Environmental Regulation 1999 Berlin: Springer 311 330

[B14] Pankratz MJ Jäckle H Bate M, Martinez Arias A Blastoderm segmentation The development of Drosophila melanogaster 1993 Cold Spring Harbor: Cold Spring Harbor Laboratory Press 467 516

[B15] Damen WGM Evolutionary conservation and divergence of the segmentation process in arthropods Dev Dyn 2007 236 1379 1391 10.1002/dvdy.21157 17440988

[B16] Lewis EB A gene complex controlling segmentation in *Drosophila* Nature 1978 276 565 570 10.1038/276565a0 103000

[B17] Struhl G Genes controlling segmental specification in the *Drosophila* thorax Proc Natl Acad Sci USA 1982 79 7380 7384 10.1073/pnas.79.23.7380 6961417 PMC347343

[B18] Lawrence PA Morata G Homeobox genes: their function in *Drosophila* segmentation and pattern formation Cell 1994 78 181 189 10.1016/0092-8674(94)90289-5 7913879

[B19] Akam M Hox genes, homeosis and the evolution of segment identity: no need for hopeless monsters Int J Dev Biol 1998 42 445 451 9654030

[B20] Pultz MA Diederich RJ Cribbs DL Kaufman TC The proboscipedia locus of the Antennapedia complex: a molecular and genetic analysis Genes Dev 1988 2 901 920 10.1101/gad.2.7.901 2850265

[B21] Hughes CL Kaufman TC RNAi analysis of *Deformed*, *proboscipedia* and *Sex combs reduced* in the milkweed bug *Oncopeltus fasciatus*: novel roles for Hox genes in the hemipteran head Development 2000 127 3683 3694 10934013 10.1242/dev.127.17.3683

[B22] Pavlopoulos A Kontarakis Z Liubicich DM Serano JM Akam M Patel NH Averof M Probing the evolution of appendage specialization by Hox gene misexpression in an emerging model crustacean Proc Natl Acad Sci USA 2009 106 13897 13902 10.1073/pnas.0902804106 19666530 PMC2728992

[B23] Khadjeh S Turetzek N Pechmann M Schwager EE Wimmer EA Damen WG Prpic NM Divergent role of the Hox gene Antennapedia in spiders is responsible for the convergent evolution of abdominal limb repression Proc Natl Acad Sci USA 2012 109 4921 4926 10.1073/pnas.1116421109 22421434 PMC3323954

[B24] Akam M Arthropods: developmental diversity within a (super) phylum Proc Natl Acad Sci USA 2000 97 4438 4441 10.1073/pnas.97.9.4438 10781039 PMC34317

[B25] Damen WGM Hausdorf M Seyfarth E-A Tautz D A conserved mode of head segmentation in arthropods revealed by the expression pattern of Hox genes in a spider Proc Natl Acad Sci USA 1998 95 10665 10670 10.1073/pnas.95.18.10665 9724761 PMC27952

[B26] Cook CE Smith LM Telford MJ Bastianello A Akam M Hox genes and the phylogeny of the arthropods Curr Biol 2001 11 759 763 10.1016/S0960-9822(01)00222-6 11378385

[B27] Hughes CL Kaufman TC Hox genes and the evolution of the arthropod body plan Evol Dev 2002 4 459 499 10.1046/j.1525-142X.2002.02034.x 12492146

[B28] Rushlow C Doyle H Hoey T Levine M Molecular characterization of the *zerknüllt* region of the Antennapedia gene complex in *Drosophila* Genes Dev 1987 1 1268 1279 10.1101/gad.1.10.1268 2892759

[B29] Stauber M Jackle H Schmidt-Ott U The anterior determinant *bicoid* of *Drosophila* is a derived Hox class 3 gene Proc Natl Acad Sci USA 1999 96 3786 3789 10.1073/pnas.96.7.3786 10097115 PMC22372

[B30] Jürgens G Wieschaus E Nüsslein-Volhard C Kluding H Mutations affecting the pattern of the larval cuticle in *Drosophila melanogaster*. II. Zygotic loci on the third chromosome Wilhelm Roux’s Arch Dev Biol 1984 193 283 295 10.1007/BF00848157 28305338

[B31] Stauber M Prell A Schmidt-Ott U A single *Hox3* gene with composite *bicoid* and *zerknüllt* expression characteristics in non-Cyclorrhaphan flies Proc Natl Acad Sci USA 2002 99 274 279 10.1073/pnas.012292899 11773616 PMC117551

[B32] Hughes CL Liu PZ Kaufman TC Expression patterns of the rogue Hox genes *Hox3/zen* and *fushi tarazu* in the apterygote insect *Thermobia domestica* Evol Dev 2004 6 393 401 10.1111/j.1525-142X.2004.04048.x 15509221

[B33] Damen WGM Janssen R Prpic N-P Pair rule gene orthologs in spider segmentation Evol Dev 2005 7 618 628 10.1111/j.1525-142X.2005.05065.x 16336415

[B34] Janssen R Damen WGM The ten Hox genes of the millipede *Glomeris marginata* Dev Genes Evol 2006 216 451 465 10.1007/s00427-006-0092-5 16816968

[B35] Papillon D Telford MJ Evolution of *Hox3* and *ftz* in arthropods: insights from the crustacean *Daphnia pulex* Dev Genes Evol 2007 217 315 322 10.1007/s00427-007-0141-8 17310351

[B36] Sharma PP Schwager EE Extavour CG Giribet G Hox gene expression in the harvestman *Phalangium opilio* reveals divergent patterning of the chelicerate opisthosoma Evol Dev 2012 14 450 463 10.1111/j.1525-142X.2012.00565.x 22947318

[B37] Whitington PM Mayer G The origins of the arthropod nervous system: insights from the Onychophora Arthropod Struct Dev 2011 40 193 209 10.1016/j.asd.2011.01.006 21315833

[B38] Hering L Henze MJ Kohler M Kelber A Bleidorn C Leschke M Nickel B Meyer M Kircher M Sunnucks P Mayer G Opsins in Onychophora (velvet worms) suggest a single origin and subsequent diversification of visual pigments in arthropods Mol Biol Evol 2012 29 3451 3458 10.1093/molbev/mss148 22683812

[B39] Eriksson BJ Fredman D Steiner G Schmid A Characterisation and localization of the opsin protein repertoire in the brain and retinas of a spider and an onychophoran BMC Evol Biol 2013 13 186 10.1186/1471-2148-13-186 24010579 PMC3851285

[B40] Eriksson BJ Tait NN Budd GE Janssen R Akam M Head patterning and *Hox* gene expression in an onychophoran and its implications for the arthropod head problem Dev Genes Evol 2010 220 117 122 10.1007/s00427-010-0329-1 20567844

[B41] Janssen R Budd GE Deciphering the onychophoran ‘segmentation gene cascade’: gene expression reveals limited involvement of pair rule gene orthologs in segmentation, but a highly conserved segment polarity gene network Dev Biol 2013 382 224 234 10.1016/j.ydbio.2013.07.010 23880430

[B42] Thompson JD Gibson TJ Plewniak F Jeanmougin F Higgins DG The CLUSTAL_X windows interface: flexible strategies for multiple sequence alignment aided by quality analysis tools Nucleic Acids Res 1997 25 4876 4882 10.1093/nar/25.24.4876 9396791 PMC147148

[B43] Henikoff S Henikoff JG Amino acid substitution matrices from protein blocks Proc Natl Acad Sci USA 1992 89 10915 10919 10.1073/pnas.89.22.10915 1438297 PMC50453

[B44] Le SQ Gascuel O An improved general amino acid replacement matrix Mol Biol Evol 2008 25 1307 1320 10.1093/molbev/msn067 18367465

[B45] Guindon S Gascuel O A simpel, fast, and accurate algorithm to estimate large phylogenies by maximum likelihood Syst Biol 2001 52 696 704 14530136 10.1080/10635150390235520

[B46] Janssen R Budd GE Gene expression suggests conserved aspects of *Hox* gene regulation in arthropods and provides additional support for monophyletic Myriapoda EvoDevo 2010 1 4 10.1186/2041-9139-1-4 20849647 PMC2938723

[B47] Grenier JK Garber TL Warren R Whitington PM Carroll S Evolution of the entire arthropod Hox gene set predated the origin and radiation of the onychophoran/arthropod clade Curr Biol 1997 7 547 553 10.1016/S0960-9822(06)00253-3 9259556

[B48] Shippy TD Rogers CD Beeman RW Brown SJ Denell RE The *Tribolium castaneum* ortholog of *Sex combs reduced* controls dorsal ridge development Genetics 2006 74 297 307 16849608 10.1534/genetics.106.058610 PMC1569817

[B49] Shultz JW Regier JC Phylogenetic analysis of arthropods using two nuclear protein-encoding genes supports a crustacean + hexapod clade Proc Biol Sci 2000 267 1011 1019 10.1098/rspb.2000.1104 10874751 PMC1690640

[B50] Regier JC Shultz JW Kambic RE Pancrustacean phylogeny: hexapods are terrestrial crustaceans and maxillopods are not monophyletic Proc Biol Sci 2005 272 395 401 10.1098/rspb.2004.2917 15734694 PMC1634985

[B51] von Reumont BM Jenner RA Wills MA Dell’ampio E Pass G Ebersberger I Meyer B Koenemann S Iliffe TM Stamatakis A Niehuis O Meusemann K Misof B Pancrustacean phylogeny in the light of new phylogenomic data: support for Remipedia as the possible sister group of Hexapoda Mol Biol Evol 2012 29 1031 1045 10.1093/molbev/msr270 22049065

[B52] Rota-Stabelli O Campbell L Brinkmann H Edgecombe GD Longhorn SJ Peterson KJ Pisani D Philippe H Telford MJ A congruent solution to arthropod phylogeny: phylogenomics, microRNAs and morphology support monophyletic Mandibulata Proc Biol Sci 2011 278 298 306 10.1098/rspb.2010.0590 20702459 PMC3013382

[B53] Sombke A Lipke E Kenning M Müller CH Hansson BS Harzsch S Comparative analysis of deutocerebral neuropils in Chilopoda (Myriapoda): implications for the evolution of the arthropod olfactory system and support for the Mandibulata concept BMC Neurosci 2012 13 1 17 10.1186/1471-2202-13-1 22214384 PMC3320525

[B54] Hughes CL Kaufman TC Exploring the myriapod body plan: expression patterns of the ten Hox genes in a centipede Development 2002 19 1225 1238 11874918 10.1242/dev.129.5.1225

[B55] Abzhanov A Popadic A Kaufman TC Chelicerate Hox genes and the homology of arthropod segments Evol Dev 1999 1 77 89 10.1046/j.1525-142x.1999.99014.x 11324031

[B56] Telford MJ Thomas RH Of mites and zen: expression studies in a chelicerate arthropod confirm *zen* is a divergent Hox gene Dev Genes Evol 1998 208 591 594 10.1007/s004270050219 9811978

[B57] Grbic M Van Leeuwen T Clark RM Rombauts S Rouze P Grbic V Osborne EJ Dermauw W Ngoc PC Ortego F Hernandez-Crespo P Diaz I Martinez M Navajas M Sucena E Magalhaes S Nagy L Pace RM Djuranovic S Smagghe G Iga M Christiaens O Veenstra JA Ewer J Villalobos RM Hutter JL Hudson SD Velez M Yi SV Zeng J The genome of *Tetranychus urticae* reveals herbivorous pest adaptations Nature 2011 479 487 492 10.1038/nature10640 22113690 PMC4856440

[B58] Aboobaker A Blaxter M Hox gene evolution in nematodes: novelty conserved Curr Opin Genet Dev 2003 13 593 598 10.1016/j.gde.2003.10.009 14638320

[B59] Martin-Duran JM Janssen R Wennberg S Budd GE Hejnol A Deuterostomic development in the protostome *Priapulus caudatus* Curr Biol 2012 22 2161 2166 10.1016/j.cub.2012.09.037 23103190

[B60] Schwager EE Schoppmeier M Pechmann M Damen WG Duplicated Hox genes in the spider *Cupiennius salei* Front Zool 2007 4 1 10.1186/1742-9994-4-1 17355624 PMC1838909

[B61] Mann RS The specificity of homeotic gene function Bioessays 1995 17 855 863 10.1002/bies.950171007 7487967

[B62] Mahaffey JW Assisting Hox proteins in controlling body form: are there new lessons from flies (and mammals)? Curr Opin Genet Dev 2005 15 422 429 10.1016/j.gde.2005.06.009 15979870

[B63] Joshi R Sun L Mann R Dissecting the functional specificities of two Hox proteins Genes Dev 2010 24 1533 1545 10.1101/gad.1936910 20634319 PMC2904943

[B64] Reed HC Hoare T Thomsen S Weaver TA White RA Akam M Alonso CR Alternative splicing modulates Ubx protein function in *Drosophila melanogaster* Genetics 2010 184 745 775 10.1534/genetics.109.112086 20038634 PMC2845342

[B65] Castelli-Gair J Akam M How the Hox gene Ultrabithorax specifies two different segments: the significance of spatial and temporal regulation within metameres Development 1995 121 2973 2982 7555723 10.1242/dev.121.9.2973

[B66] Castelli-Gair J Implications of the spatial and temporal regulation of Hox genes on development and evolution Int J Dev Biol 1998 42 437 444 9654029

[B67] Smolik-Utlaut SM Dosage requirements of Ultrabithorax and bithoraxoid in the determination of segment identity in *Drosophila melanogaster* Genetics 1990 124 357 366 1968411 10.1093/genetics/124.2.357 PMC1203927

[B68] Tour E Hittinger CT McGinnis W Evolutionarily conserved domains required for activation and repression functions of the *Drosophila* Hox protein Ultrabithorax Development 2005 132 5271 5281 10.1242/dev.02138 16284118

[B69] Liubicich DM Serano JM Pavlopoulos A Kontarakis Z Protas ME Kwan E Chatterjee S Tran KD Averof M Patel NH Knockdown of *Parhyale Ultrabithorax* recapitulates evolutionary changes in crustacean appendage morphology Proc Natl Acad Sci USA 2009 106 13892 13896 10.1073/pnas.0903105106 19666517 PMC2720850

