# Supplementary figures and images for: Onychophoran Hox genes and the evolution of arthropod Hox gene expression
Source: Front Zool. 2014 Mar 5;11:22. doi: 10.1186/1742-9994-11-22 (PMC4015684; doi:10.1186/1742-9994-11-22)

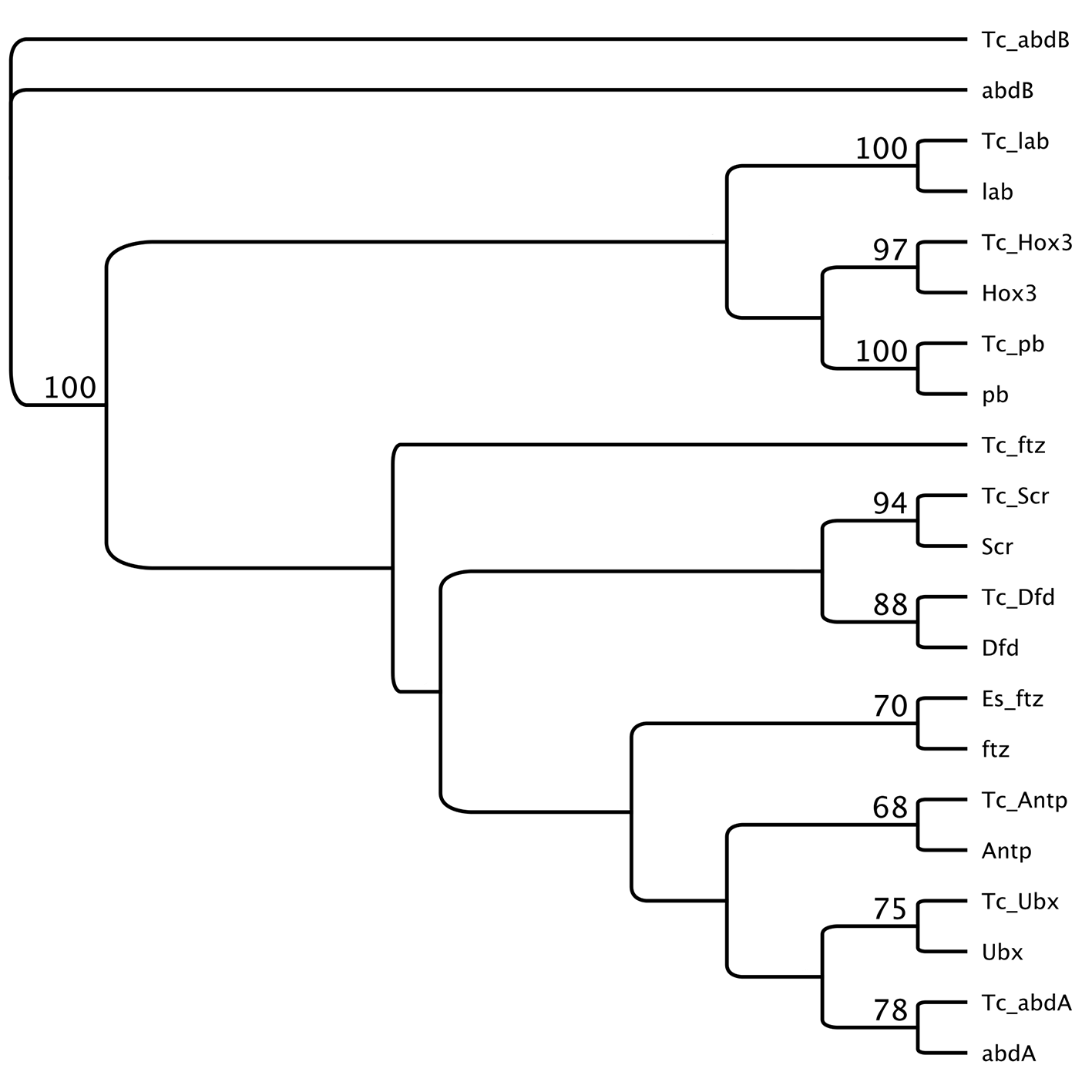

Supplement: Additional file 1: Figure S1 — Phylogenetic analysis of E. kanangrensis Hox genes. The homeodomain sequences have been used for the analyses. The phylogram represents the unrooted majority rule consensus computed from 100 replicas. Numbers indicate the reliability values of the branches. [file 1742-9994-11-22-S1.tiff]

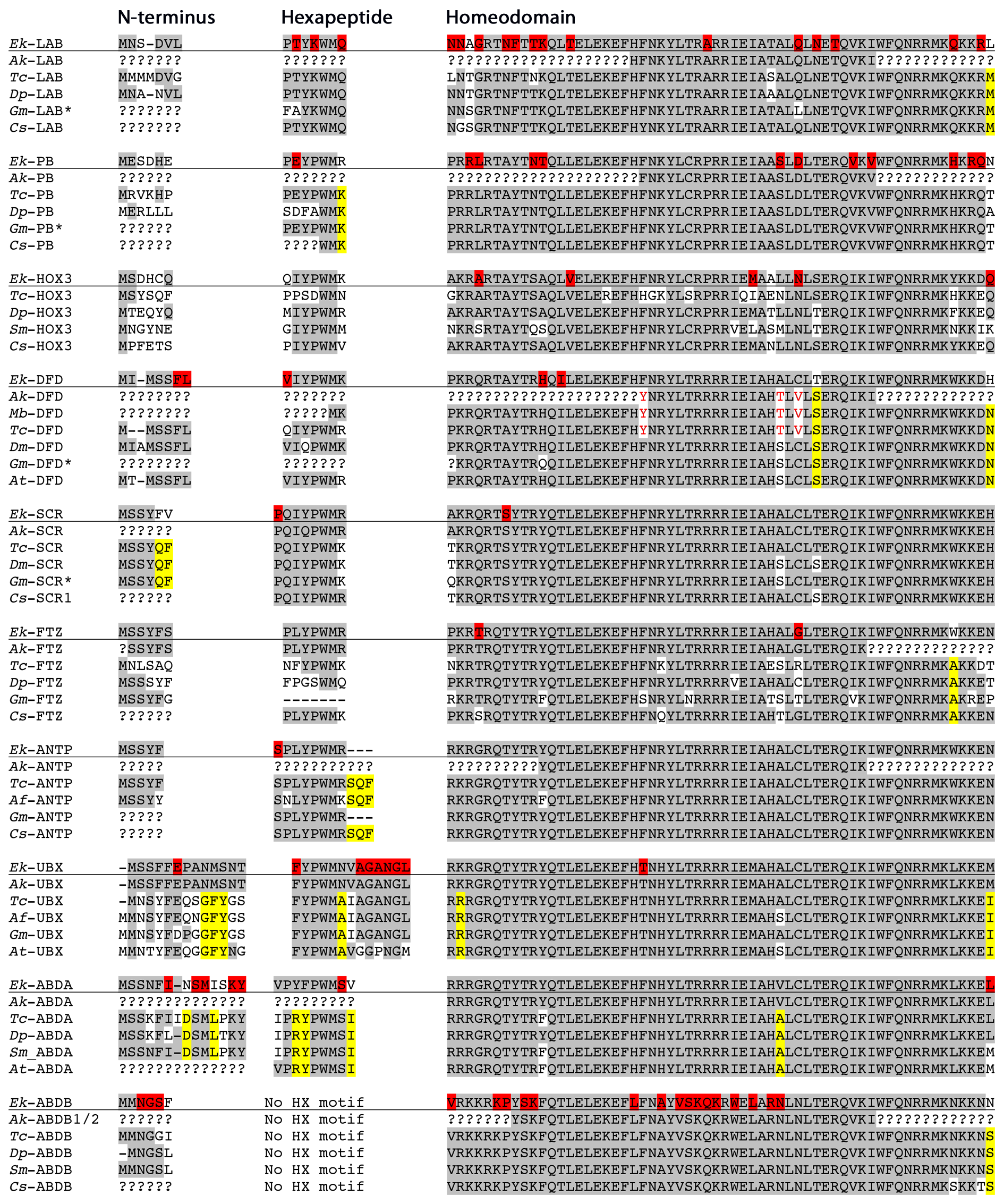

Supplement: Additional file 2: Figure S2 — Conserved protein motifs in arthropod and onychophoran Hox genes. Onychophoran Hox sequences (E. kanangrensis and Akanthokara kaputensis) are aligned with orthologous genes of at least one species representing the four main arthropod classes (i.e. Insecta, Crustacea, Myriapoda and Chelicerata). Identical amino acids (compared to E. kanangrensis) are shaded in grey; amino acids that are conserved in arthropods are shaded in yellow; diagnostic amino acids are shaded in red. Amino acids in red font indicate insect-specific positions (see text for further information). Question marks (?) stand for unknown sequence. Dashes (-) indicate the absence of any amino acid in this position of the alignment. Sequences of E. kanangrensis are underlined. Asterisks (*) mark G. marginata sequences that are partially recovered from an embryonic transcriptome. Abbreviations: Af, Artemia franciscana (Crustacea); Ak, Akanthokara kaputensis (Onychophora); At, Achaearanea tepidariorum (Chelicerata); Cs, Cupiennius salei (Chelicerata); Dm, Daphnia magna (Crustacea) Dp, Daphnia pulex (Crustacea); Ek, Euperipatoides kanangrensis (Onychophora); Glomeris marginata (Myriapoda); Mb, Mamestra brassicae (Insecta); Sm, Strigamia maritima (Myriapoda); Tc, Tribolium castaneum (Insecta). [file 1742-9994-11-22-S2.tiff]

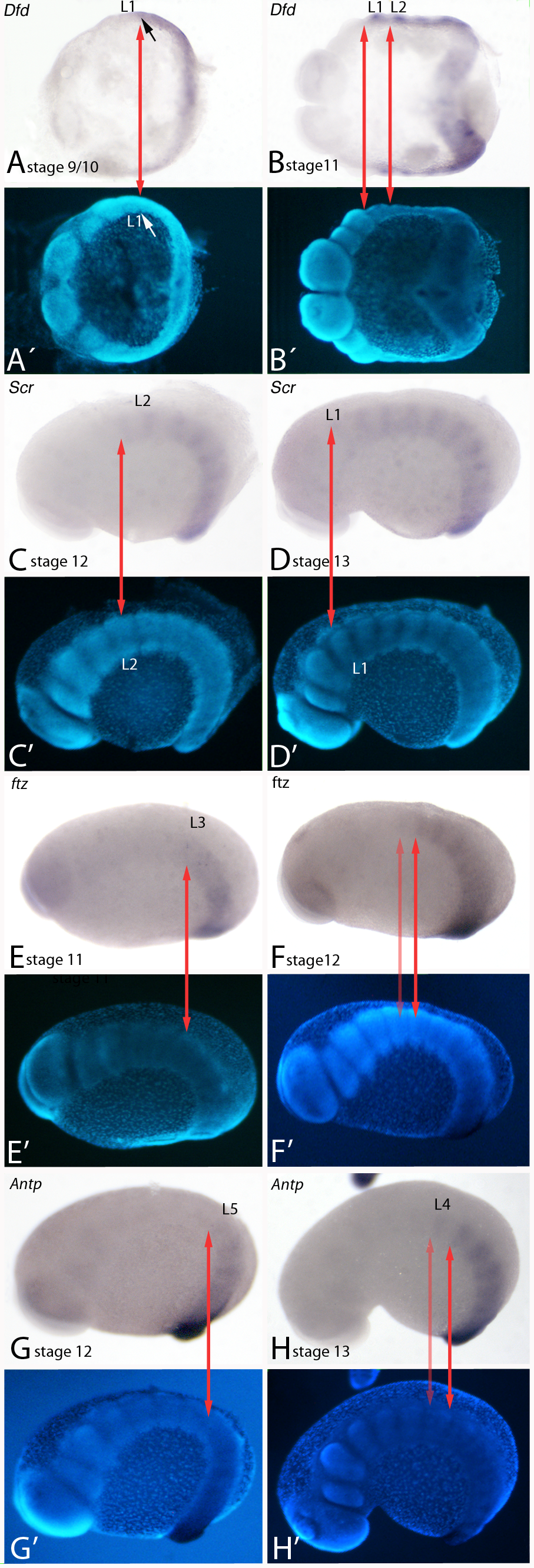

Supplement: Additional file 3: Figure S3 — Shifted anterior borders of Hox gene expression. All embryos are oriented with anterior to the left. A/B: Ventral view. C-H: lateral view. A-H) bright field photographs; A’-H’) DAPI staining of the same embryos shown in A-H. Double-headed arrows point to anterior border of expression. Faint arrows indicate weak expression. A) Expression of Ek-Dfd is anteriorly restricted to L2 (stage 9/10). B) At stage 11 Ek-Dfd expression extends to the anterior border of L1. C) Expression of Ek-Scr extends into the middle of L2 at stage 12. D) At stage 13 Ek-Scr shifts towards anterior into the L1-segment. E) Anterior border of expression of Ek-ftz is in the middle of L3 at stage 11. F) At the subsequent stage 12, Ek-ftz is weakly present also in L2. G) Expression of Ek-Antp is restricted to L5 (and more posterior segments) at stage 12. H) At stage 13, Ek-Antp reaches anteriorly into the L4 segment where it remains expressed weakly during further development. Shifting of the anterior border of expression was never observed for any of the other onychophoran Hox genes. Abbreviations: L1-L5, first to fifth walking limb-bearing segment. [file 1742-9994-11-22-S3.tiff]

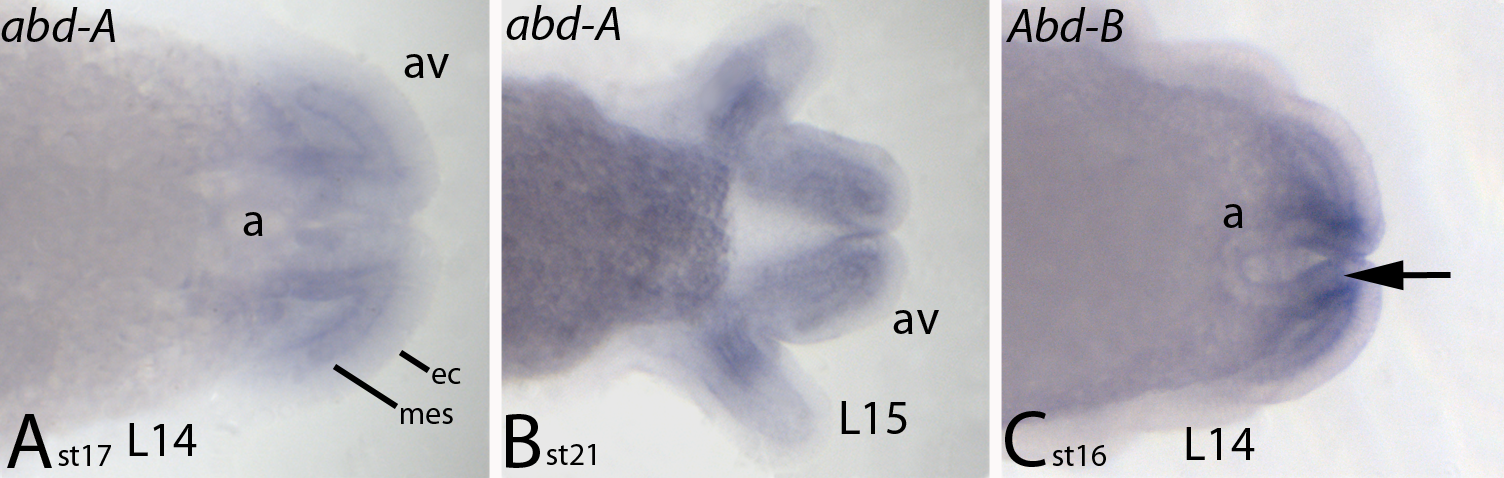

Supplement: Additional file 4: Figure S4 — Additional aspects of Ek-abd-A and Ek-Abd-B expression. In all panels anterior is to the left. A) Expression of Ek-abd-A. Ventral view. Expression in the mesoderm (mes) is stronger than in the ectoderm (ec). B) Expression of Ek-abd-A; dorsal view. C) Expression of Ek-Abd-B. Ventral view. The arrow points to expression in the ectoderm of the anal valves. Abbreviations: a, anus; av, anal valve; ec, ectoderm; mes, mesoderm; L14 and L15, fourteenth and fifteenth walking-limb bearing segment. [file 1742-9994-11-22-S4.tiff]

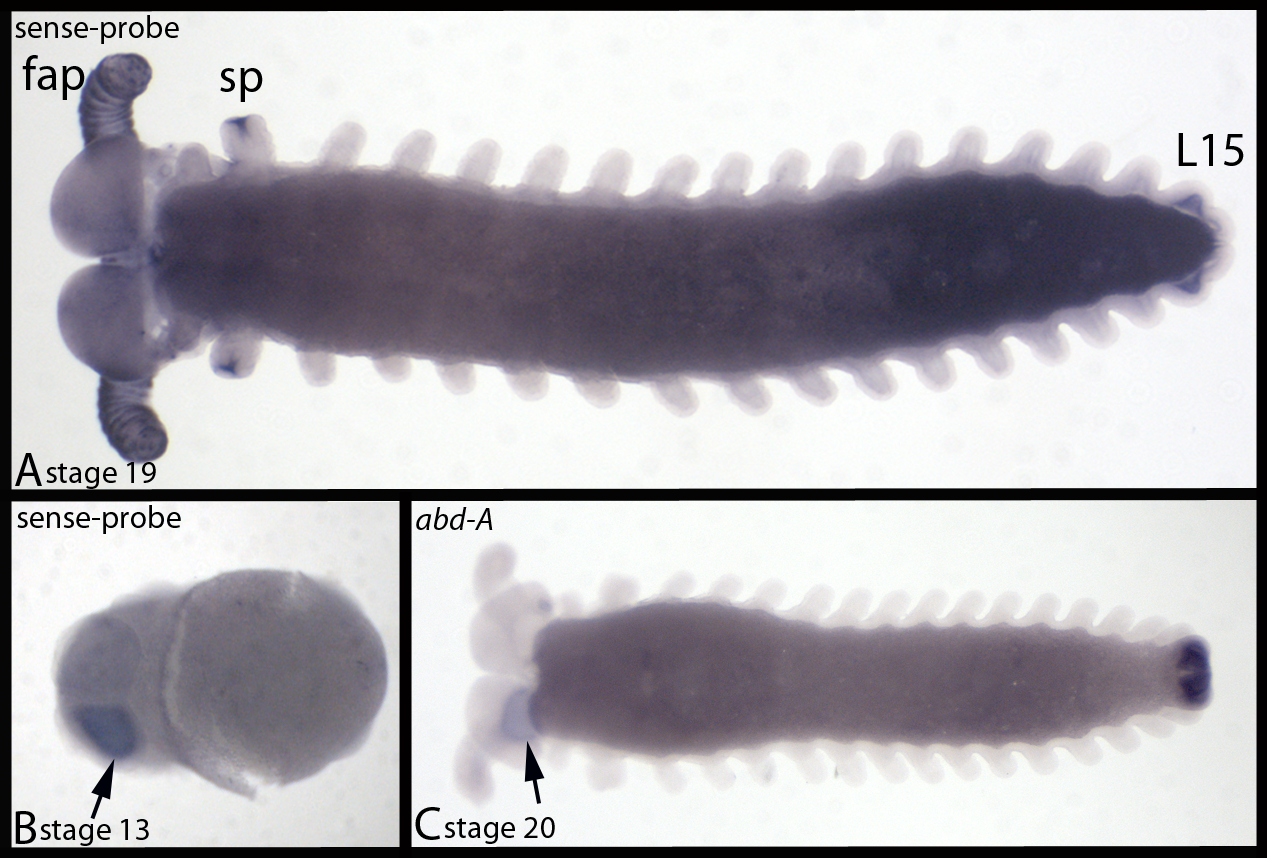

Supplement: Additional file 5: Figure S5 — Frequently occurring staining artefacts. In all panels anterior is to the left. A) Stage 19 embryo. Ventral view. Typical staining artefacts are often observed in older stage embryos where the surface of the frontal appendages attracts unspecific signal. A similar false positive signal appears regularly in the opening of the slime papillae. The coelomic cavity of the L15-segment often stains unspecifically. Long staining time frequently results in unspecific staining of the yolk. B/C) A typical artefact: staining of one of the two coelomic cavities (arrowheads) of the head in a stage 13 and a stage 20 embryo. Abbreviations: fap, frontal appendage; L15, fifteenth walking limb; sp, lime papilla. [file 1742-9994-11-22-S5.tiff]
