# Supplementary material for: Onychophoran Hox genes and the evolution of arthropod Hox gene expression
Source: Front Zool. 2014 Mar 5;11:22. doi: 10.1186/1742-9994-11-22 (PMC4015684; doi:10.1186/1742-9994-11-22)
Supplement: Additional file 6: Table S1 — Summary of available literature on Hox gene expression in arthropods and onychophorans. Compared is the anterior-most extension of Hox gene mRNA (in some cases protein) expression. [file 1742-9994-11-22-S6.doc]

| Gene | Conserved anterior border of expression | Arthropod class | References in support for the presented anterior expression border | | References in support for deviant anterior expression border |
| --- | --- | --- | --- | --- | --- |
| *lab* | H1 | Onychophora | Eriksson et al. 2010 | | NONE |
|  | H1 | Chelicerata | Damen et al. 1998, Jager et al. 2006, Sharma et al. 2012, Khadjeh et al. 2012 | | NONE |
|  | H1 | Myriapoda | Hughes and Kaufman 2002b, Janssen and Damen 2006 | | NONE |
|  | H1 | Crustacea | Abzhanov and Kaufman 1999a | | NONE |
|  | H1 | Insecta | Diederich et al. 1989, Peterson et al. 1999, Nie et al., 2001 | | NONE |
| *pb* | H1 | Onychophora | Eriksson et al. 2010 | | NONE |
|  | H1 | Chelicerata | Telford and Thomas 1998b, Abzhanov et al. 1999, Jager et al. 2006, Schwager et al. 2007, Sharma et al. 2012 | | NONE |
|  | H1 | Myriapoda | Hughes and Kaufman 2002b, Janssen and Damen 2006 | | NONE |
|  | H1 | Crustacea | Abzhanov and Kaufman 1999a | | NONE |
|  | Different  (H1 or H2) | Insecta | Shippy et al. 2000, Rogers et al. 2002 | | Liu and Kaufman 2004 (H2), Rogers et al. 2002 (H2), Angelini et al. 2005 (H2), Randazzo et al. 1991 (H2) |
| *Hox3* | H1 | Onychophora | Eriksson et al. 2010 | | NONE |
|  | H1 | Chelicerata | Telford and Thomas 1998b, Damen and Tautz 1998, Abzhanov et al. 1999, Sharma et al. 2012 | | NONE |
|  | H1 | Myriapoda | Hughes and Kaufman 2002b, Janssen and Damen 2006 | | NONE |
|  | H1 | Crustacea | Papillon and Telford 2007 | | NONE |
|  | Different  (H1, H2, or non-Hox expression) | Insecta | NONE | | Hughes et al. 2004 (H2), Diverged function/expression in ‘higher’ insects |
| *Dfd* | H2 | Onychophora | Eriksson et al. 2010 | | NONE |
|  | H2 | Chelicerata | Damen et al. 1998, Telford and Thomas 1998b, Abzhanov et al. 1999, Jager et al. 2006, Schwager et al. 2007, Sharma et al. 2012, Khadjeh et al. 2012 | | NONE |
|  | H2 | Myriapoda | Hughes and Kaufman 2002b, Janssen and Damen 2006 | | NONE |
|  | H2 | Crustacea | Abzhanov and Kaufman 1999a | | NONE |
|  | H2 | Insecta | Chadwick and McGinnis 1987, Walldorf et al. 2000, Kokubo et al. 1997, Brown et al. 1999, Rogers et al. 2002 | | NONE |
| *Scr* | Different (H2) | Onychophora | NONE | | THIS STUDY (H2) |
|  | H3 | Chelicerata | Telford and Thomas 1998b, Abzhanov et al. 1999, Schwager et al. 2007, Sharma et al. 2012, Khadjeh et al. 2012 | | NONE |
|  | H3 | Myriapoda | Janssen and Damen 2006, Hughes and Kaufman 2002b^(1)^ | | NONE |
|  | H3 | Crustacea | Abzhanov and Kaufman 1999b, 2000b | | NONE |
|  | H3 | Insecta | Martinez-Arias et al. 1987, Kokubo et al. 1997, Rogers et al. 1997, Walldorf et al. 2000, Curtis et al. 2001, Zhang et al. 2005 | | NONE |
| *ftz* | H3 | Onychophora | THIS STUDY | | NONE |
|  | H3 | Chelicerata | Telford 2000, Damen et al. 2005, Sharma et al. 2012 | | NONE |
|  | H3 | Myriapoda | Hughes and Kaufman 2002b, Janssen and Damen 2006 | | NONE |
|  | Different  (H2 or non-Hox expression) | Crustacea | NONE | | Mouchel-Vielh et al. 2002^(2)^, Papillon and Telford 2007 (H2) |
|  | Different  (H2 or non-Hox expression) | Insecta | NONE | | Hughes et al. 2004 (H2)  Diverged function/expression in ‘higher’ insects |
| *Antp* | H5 | Onychophora | THIS STUDY | | NONE |
|  | H5 | Chelicerata | Damen et al. 1998, Telford and Thomas 1998b, Sharma et al. 2012 | | NONE |
|  | H5 | Myriapoda | Janssen and Damen 2006^(3)^ | | Hughes and Kaufman 2002b (H4)^(1)^, Brena et al. 2006 (H4) |
|  | Different (H4) | Crustacea | NONE | | Abzhanov and Kaufman 2000a (H4), Abzhanov and Kaufman 2000b (H4), Averof and Akam 1995 (H3), Shiga et al. 2006 (H4), Zhang et al. 2005 (H4) |
|  | Different (H4) | Insecta | NONE | | Walldorf et al. 2000 (H4), Hayward et al. 1995 (H4) |
| *Ubx* | H6 (?) | Onychophora | THIS STUDY (gradient, possibly anterior border in H6) | | |
|  | H6 (basally branching chelicerates) | Chelicerata | Popadic and Nagy 2001 | Damen et al. 1998 (H7), Abzhanov et al. 1999 (H7), Sharma et al. 2012 (H7), Khadjeh et al. 2012 (H7) | |
|  | H6 | Myriapoda | Grenier et al. 1997, Abzhanov et al. 1999, Hughes and Kaufman 2002b, Janssen and Damen 2006, Brena et al. 2006 | NONE | |
|  | Variation (H4 - H8) | Crustacea | Averof and Patel 1997, Abzhanov and Kaufman 2000b, Liubicich et al. 2009 | Averof and Akam 1995 (H4), Averof and Patel 1997 (segs-5-8), Abzhanov and Kaufman 2000a (H5) | |
|  | Variation (H6 - H8) | Insecta | White and Wilcox 1985, Warren et al. 1994, Kelsh et al. 1994, Peterson et al. 1999, Zheng et al. 1999, Bennett et al. 1999, Masumoto et al. 2009, Khila et al. 2009 | Walldorf et al. 2000 -PRT (H8), Mahfooz et al. 2007 (H7), Zhang et al. 2005 (H7) | |
| *abd-A* | L15 | Onychophora | THIS STUDY | | |
|  | Variation (H7/H8) | Chelicerata | Damen et al. 1998 (H8), Sharma et al. 2012 (H7), Khadjeh et al. 2012 (H8) | | |
|  | Variation (H6/H7) | Myriapoda | Hughes and Kaufman 2002b (H6), Janssen and Damen 2006 (H7), Brena et al. 2006 (H6) | | |
|  | Variation (H6/H11) | Crustacea | Abzhanov and Kaufman 2000b (H6), Abzhanov and Kaufman 2000a (H6), Averof and Akam 1995 (H11) | | |
|  | H8 | Insecta | Warren et al. 1994 (H8), Petersson et al. 1999 (H8), Tear et al. 1990 (H8), Shippy et al., 1998 (H8), Nagy et al. 1991 (H8), Macias et al. 1990 (H8), Niculita 2006, Zhang et al. 2005 (H8), Tomita and Kiguchi 2009 (H8) | | |
| *Abd-B* | Posterior/Genital | Onychophora | THIS STUDY | NONE | |
| (posterior, genital) | Posterior/Genital | Chelicerata | Damen and Tautz 1999^(4)^ | Damen and Tautz 1999, Sharma et al. 2012 | |
|  | Posterior/Genital | Myriapoda | Janssen and Damen 2006, Brena et al. 2006 | Hughes and Kaufman 2002b | |
|  | Posterior/Genital | Crustacea | Averof and Akam 1995, Blin et al. 2003^(5)^, Brena et al. 2005^(6)^ | Blin et al. 2003^(5)^, Brena et al. 2005^(6)^ | |
|  | Posterior/Genital | Insecta | Peterson et al. 1999, Kelsh et al. 1993, Yoder and Carroll 2006, Tomita and Kikuchi 2009 | Delorenzi and Bienz 1990^(7)^ | |

^(3)^ expression could be interpreted as reaching into pmd (=seg4), problem of young stage without land marks and the lack of double in-situs with e.g. segment polarity genes as molecular markers

^(1)^ note discrepancy between text/panel and diagram for anterior border of Antp and Scr

^(2)^ This paper reports on a ftz-like gene in a cirripede crustacean, but the expression appears unlike that of a typical Hox gene.

^(4)^ referring to the patch-like expression in the genital segment

^(5)^ two populations of larva with different abd-B expression assumed to be male and female; expression in assumed genital segments, but also spanning the complete thorax and abdomen; therefore the paper is listed as support and non-support for the conserved expression posterior and/or the genital segments(s)

^(6)^ an early posterior-only expression domain, but a broad later expression throughout the pleon; therefore the paper is listed as support and non-support for the conserved expression posterior and/or the genital segment(s)

^(7)^ special function of *abdB* in Drosophila; two versions of the gene (r and m) with different expression domains
